# Intravenous Fluid Challenge Decreases Intracellular Volume: A Bioimpedance Spectroscopy-Based Crossover Study in Healthy Volunteers

**DOI:** 10.1038/s41598-017-09433-5

**Published:** 2017-08-29

**Authors:** Matthäus Ernstbrunner, Barbara Kabon, Oliver Zotti, Markus Zeitlinger, Carolin Berner, Georg Hinterholzer, Marcus Säemann, Florian Frommlet, Edith Fleischmann, Manfred Hecking

**Affiliations:** 1 Department of Anaesthesiology and Critical Care Medicine, Medical University of Vienna, Währinger Gürtel 18-20, A-1090 Vienna, Austria; 2 Department of Clinical Pharmacology, Medical University of Vienna, Währinger Gürtel 18-20, A-1090 Vienna, Austria; 3 Kaiser-Franz-Josef Spital -Sozialmedizinisches Zentrum Süd- Medical Department I, Kundratstraße 3, A-1100 Vienna, Austria; 4 Department of Internal Medicine III, Clinical Division of Nephrology & Dialysis, Medical University of Vienna, Währinger Gürtel 18-20, A-1090 Vienna, Austria; 5 Center for Medical Statistics, Informatics and Intelligent Systems, Medical University of Vienna, Währinger Gürtel 18-20, A-1090 Vienna, Austria

## Abstract

The effects of intravenous fluid therapy on fluid compartments and hemodynamics of the human body remain enigmatic. We therefore tested the efficacy of bioimpedance spectroscopy in a crossover study, where 15 males received 0.5 ml/kg/min ELO-MEL-isoton (osmolarity = 302 mosmol/l) during 60 minutes, or nothing at all. In group “Fluid”, fluid load increased from −0.2 ± 1.0 l extracellular volume at baseline to its maximum of 1.0 ± 0.9 l in minute 70, and remained continuously elevated throughout minute 300. In group “Zero”, fluid load decreased from 0.5 ± 1.1 l at baseline to its minimum of −1.1 ± 1.1 l in minute 300. In group “Fluid”, intracellular volume decreased from 26.8 ± 3.9 l at baseline to its minimum of 26.0 ± 3.9 l in minute 70, and remained continuously decreased throughout minute 300. In group “Zero”, intracellular volume increased from 26.5 ± 3.8 l at baseline to its maximum of 27.1 ± 3.9 l in minute 120, and decreased thereafter. In group “Fluid” compared to “Zero”, systolic blood pressure was significantly higher, from minute 50–90. In conclusion, intravenous fluid therapy caused a clinically meaningful, sustained increase in fluid load, and a decrease in intracellular volume. These data raise interest in studying fluid administration by the gastrointestinal route, perhaps even when managing critical illness.

## Introduction

Interfering with the human fluid volume status by administering an intravenous (iv) infusion solution routinely concerns any medical discipline today^1, 2^, but represents a considerable therapeutic challenge^3–5^. Essential decisions, such as amount of infusion and fluid type to be administered^6^ are frequently made in absence of clinical knowledge on the desired ranges of daily electrolyte supply and urinary output^5^. Even for medical experts, fluid volume assessment, which essentially means determining if an individual might be fluid overloaded or dehydrated, is a difficult task, particularly in the presence of disease^7^.

Whole-body bioimpedance spectroscopy using the Body Composition Monitor (BCM) has been extensively validated^8–10^ and aids fluid volume assessment by providing numerical values for total body volume, intracellular and extracellular volume, as well as for fluid load (termed fluid overload on the BCM-device, in litres and % extracellular volume), based on a three-compartment physiologic tissue model^8^. In our previous non-interventional, observational cohort study on 71 females undergoing gynaecological procedures, we have analysed the results of blinded BCM-measurements before and after general anaesthesia^11^. Perioperatively, we observed a significant increase in fluid load, extracellular volume and total body volume, amounting to roughly half of the routinely administered infusion solution^11^.

Various elements like the systemic stress response^12^ and local inflammation^13^ resulting in capillary leak^14^ may influence the perioperative fluid distribution. However, as all subjects in our prior cohort had normal renal function, we were surprised to detect a clinically meaningful fluid shift at all^11^. A comprehensive search of the literature revealed that the immediate and intermediate effects of intravenous infusion therapy on the fluid compartments of the human body, as well as the fluid volume ‘status quo’ before the start of any intravenous therapy, are not routinely captured in medical practice. Although fluid therapy is a common medical task^15^, its basic physiological consequences may thus be largely unknown to medical care takers.

Bioimpedance spectroscopy can easily be put into practice. The present cross-over study in healthy volunteers was designed to test the efficacy of whole-body bioimpedance spectroscopy for assessment of fluid volume status at baseline, as well as for detection of rapid and longer-term changes in fluid load, extracellular volume, intracellular volume and total body volume (if any), under standardized intravenous fluid therapy. As a secondary objective, we aimed at recording the potential changes in hemodynamic parameters: blood pressure, peripheral capillary oxygen saturation and saturated and heart rate.

## Results

**Figure 1:**
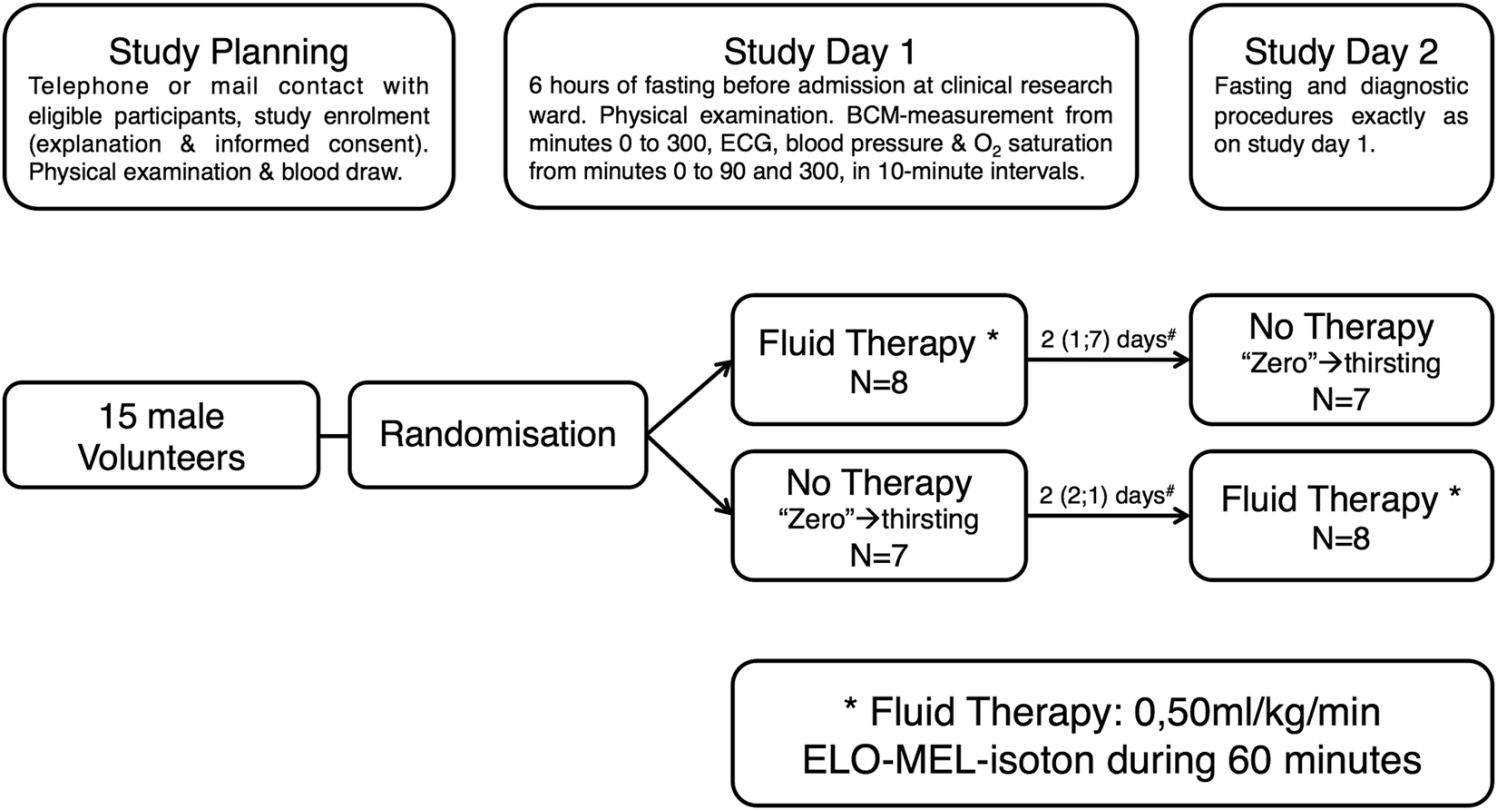
Study flow chart. ^#^Values are reported as median (1^st^ quartile; 3^rd^ quartile). N = number of participants.

The 15 study participants were healthy males between 22 and 41 years of age (29.3 ± 5.3 years on average ± standard deviation), with body mass index 23.1 ± 3.0 kg/m^2^, normal renal function and normal hepatic function, and absence of other laboratory value abnormalities (Table 1). After randomization, 8 participants received fluid therapy on study day 1 and switched to no therapy on study day 2, while 7 participants received no therapy on study day 1 and switched to fluid therapy on study day 2. The median time between study day 1 and study day 2 was 2 days (Fig. 1). All participants completed the study. Irrespective of the study day, BCM-derived measurements and hemodynamic parameters at all time points were analysed in two groups, namely study group “Fluid” (N = 15 participants) and study group “Zero” (N = 15 participants). In study group “Fluid”, the participants received 2.2 ± 0.2 l ELO-MEL-isoton infusion solution within 63.8 ± 1.8 minutes, while in study group “Zero” the participants remained thirsting.

**Table 1:** Demographics and clinical characteristics of the study population.

N = 15 males	
Age [years]	29.3 ± 5.3
Height [cm]	182.7 ± 8.5
Body weight [kg]﻿	77.4 ± 12.7
Body mass index [kg/m^2^]	23.1 ± 3.0
ASA 1	15 (100%)
NYHA 1	15 (100%)
Serum Creatinine [mg/dl]	0.9 ± 0.1
γ- Glutamyl Transferase [U/l]	17.3 ± 8.8
Serum Protein [g/l]	70.9 ± 3.9
Serum Albumin [g/l]	45.3 ± 1.8
Haemoglobin [mg/dl]	14.9 ± 0.6
C-reactive Protein [mg/dl]	0.09 ± 0.1
Capillary Leak Index [unitless]	0.2 ± 0.3
Colloidosmotic Pressure [mmHg]	26.5 ± 2.4

All continuous variables are reported as means ± standard deviations. Categorical variables are presented as counts and frequencies. ASA = American Society of Anaesthesiologists physical status classification system. NYHA = New York Heart Association functional classification.

### BCM-Derived Measurements

#### Fluid load, extracellular volume and relative fluid load

**Figure 2:**
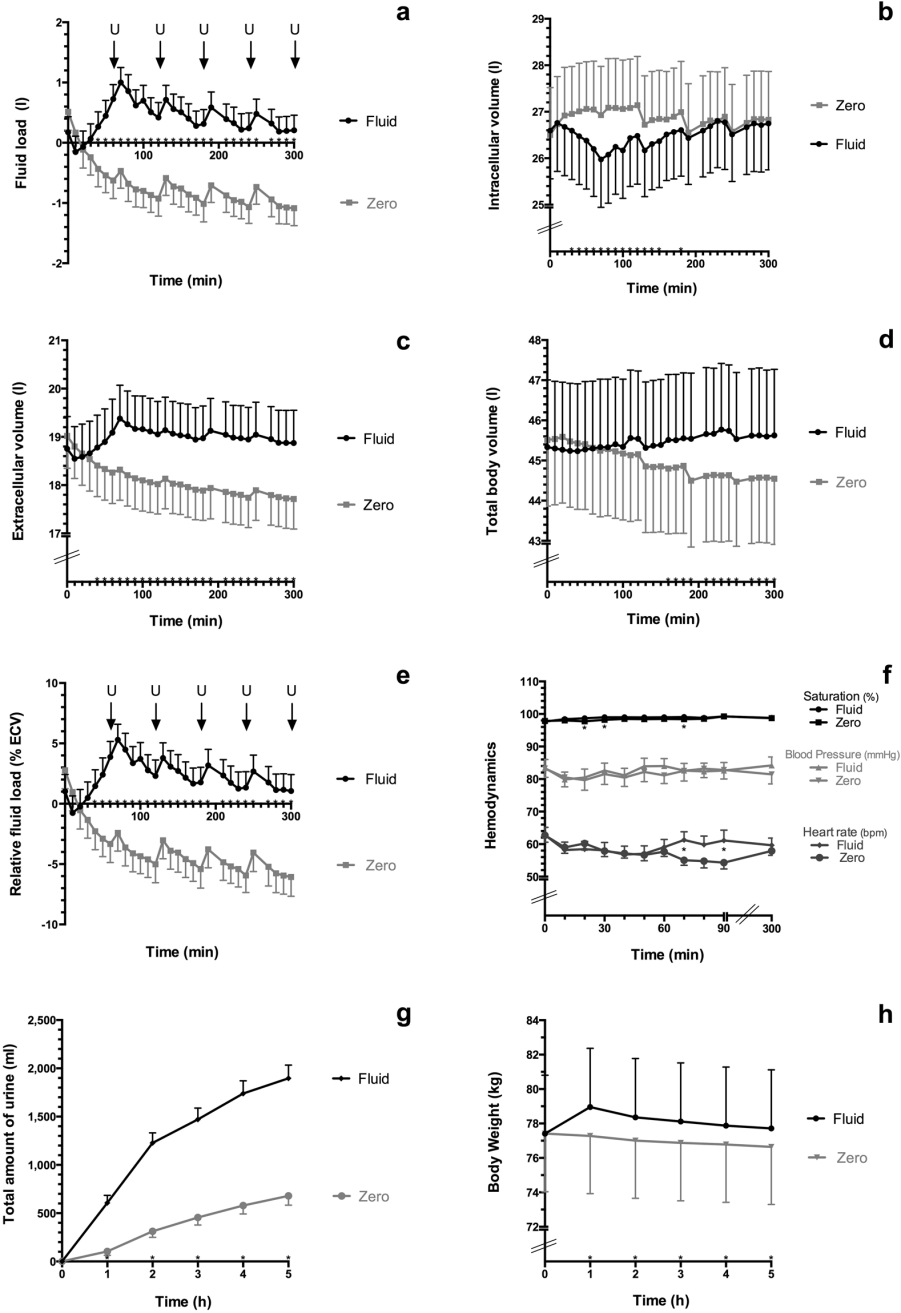
Volume status, hemodynamics, urine output and body weight during the fluid intervention (“Fluid”) and thirsting (“Zero”). Time course of fluid load (**a**), intracellular volume (**b**), extracellular volume (**c**), total body volume (**d**), relative fluid load (**e**), hemodynamics (**f**), urinary excretion (**g**) and body weight (**h**). Values are depicted as means ± standard errors of the means. Significant differences were determined by Student’s t-test and are marked (black stars) at the 0.05 probability level. U marks time point of urinating in an upright position.

In study group “Fluid”, fluid load increased from the baseline value of −0.2 ± 1.0 l to the maximal value of 1.0 ± 0.9 l in minute 70 and remained continuously elevated in comparison to baseline throughout minute 300. In study group “Zero”, fluid load decreased from the baseline value of 0.5 ± 1.1 l in minute 0 to the minimal value of −1.1 ± 1.1 l in minute 300 (Fig. 2a, Table 2). In study group “Fluid”, extracellular volume increased from the baseline value of 18.5 ± 2.5 l to the maximal value of 19.4 ± 2.6 l in minute 70 and remained continuously elevated in comparison to baseline, throughout minute 300 (Fig. 2c, Table 2). In study group “Zero”, extracellular volume decreased from the baseline value of 19.0 ± 2.5 l to the minimal value of 17.7 ± 2.3 l in minute 300 (Fig. 2c, Table 2). Relative fluid load (in % extracellular volume) changed accordingly (Fig. 2e, Table 2).

**Table 2:** BCM-derived fluid volume measurements during the fluid intervention (“Fluid”) and thirsting (“Zero”).

	Fluid Load [l]		Relative Fluid Load [% extracellular volume]		Total Body Volume [l]		Extracellular Volume [l]		Intracellular Volume [l]	
	Fluid	Zero	Fluid	Zero	Fluid	Zero	Fluid	Zero	Fluid	Zero
Baseline^†^	−0.2 ± 1.0	0.5 ± 1.1	−0.8 ± 5.3	2.8 ± 5.7	45.3 ± 6.3	45.5 ± 6.2	18.5 ± 2.5	19.0 ± 2.5	26.8 ± 3.9	26.5 ± 3.8
Minimum°	−0.2 ± 1.0	−1.1 ± 1.1	−0.8 ± 5.3	−6.1 ± 6.0	45.2 ± 6.3	44.5 ± 6.1	18.5 ± 2.5	17.7 ± 2.3	26.0 ± 3.9	26.5 ± 3.8
Maximum°	1.0 ± 0.9	0.5 ± 1.1	5.3 ± 4.8	2.8 ± 5.7	45.8 ± 6.2	45.6 ± 6.2	19.4 ± 2.6	19.0 ± 2.5	26.8 ± 3.8	27.1 ± 3.9
Mean°	0.4 ± 0.9	−0.7 ± 1.1	2.2 ± 4.9	−3.8 ± 5.6	45.5 ± 6.2	45.0 ± 6.1	19.0 ± 2.5	18.1 ± 2.4	26.5 ± 3.8	26.9 ± 3.9
Completion^§^	0.3 ± 0.9	−1.0 ± 1.1	1.5 ± 5.0	−5.5 ± 5.9	45.6 ± 6.2	44.6 ± 6.1	18.9 ± 2.5	17.8 ± 2.3	26.7 ± 3.8	26.8 ± 3.9
Δ Baseline−to-completion^#^	0.4 ± 0.6	−1.5 ± 0.6	2.3 ± 2.9	−8.2 ± 2.9	0.3 ± 0.6	−1.0 ± 0.7	0.4 ± 0.5	−1.2 ± 0.5	−0.1 ± 0.4	0.3 ± 0.5

All values are means ± standard deviations. ^†^Value obtained at 0 or 10 minutes. °Average values reported for Minimum, Maximum and Mean from Baseline to min 300. ^§^All BCM-derived fluid volume measurements from min 240 to min 300 (during the last study hour) were averaged. ^#^Mean difference (delta) from baseline to completion.

#### Intracellular volume

In study group “Fluid”, intracellular volume decreased from the baseline value of 26.8 ± 3.9 l to the minimal value of 26.0 ± 3.9 l in minute 70; intracellular volume thereafter increased, but remained continuously decreased in comparison to baseline throughout minute 300 except minute 230 and 240 (Fig. 2b, Table 2). In study group “Zero”, intracellular volume increased from the baseline value of 26.5 ± 3.8 l to the maximal value of 27.1 ± 3.9 l in minute 120; intracellular volume thereafter decreased, but remained continuously elevated in comparison to baseline throughout minute 300 (Fig. 2b, Table 2).

#### Total body volume

In study group “Fluid”, total body volume increased from the baseline value of 45.3 ± 6.3 l to the maximal value of 45.8 ± 6.2 l in minute 230 and remained continuously elevated in comparison to baseline throughout minute 300 (Fig. 2d, Table 2). In study group “Zero”, total body volume decreased from the baseline value of 45.5 ± 6.2 l in minute 0 to the minimal value of 44.5 ± 6.0 l in minute 250 and remained continuously decreased in comparison to baseline throughout minute 300 (Fig. 2d, Table 2).

#### Statistical Work-up

Results from the mixed model analysis for AUC of the five studied BCM-derived variables are listed in Table 3. The fixed effect “Study Day” did not have a significant influence on any of the five BCM-derived variables, indicating that it did not matter in the crossover design on which day the treatment was given. For the fixed effect “Treatment” we obtained strongly significant differences of the AUCs between the two study groups (“Fluid” versus “Zero”) for fluid load, relative fluid load and extracellular volume (p < 0.001), which clearly remained significant even after Bonferroni correction. Intracellular volume was borderline significant (p = 0.052) at the uncorrected level alpha = 0.05, whereas total body volume was not significant but still had a fairly small p-value of p = 0.083. Regarding total body volume, Fig. 2d illustrates that both groups behaved similarly during the first 120 minutes, while thereafter, the values for study group “Zero” started to become smaller than the values for study group “Fluid”. Using the AUC for the last 180 minutes of the experiment (starting after 120 minutes) provided a significant difference (p = 0.014). Similarly, when using only the first 180 minutes for intracellular volume, the AUC between the two study groups differed significantly (p = 0.0061), due to a marked decrease in the study group “Fluid” and an increase in the study group “Zero” (Fig. 2b).

**Table 3:** Area under the curve (AUC) of repeated BCM-derived fluid volume measurements, during the fluid intervention (“Fluid”) and thirsting (“Zero”).

	Fluid	Zero	
Fluid Load [AUC]	121.1 ± 271.3	−215.2 ± 317.3	p < 0.001**
Relative Fluid Load [AUC]	663.9 ± 1457.5	−1,164.7 ± 1674.7	p < 0.001**
Total Body Volume [AUC]	13,642 ± 1862	13,483 ± 1841	p = 0.083
Extracellular Volume [AUC]	5,695.4 ± 756.6	5,421.9 ± 708.2	p < 0.001**
Intracellular Volume [AUC]	7,947.2 ± 1140.6	8,060.7 ± 1168.0	p = 0.052

All values are means ± standard deviations. **Significant difference between “Fluid “and “Zero” was determined by Student’s t-test at the 0.01 probability level.

### Urinary Volume Excretion, Body Weight and Hemodynamic

In study group “Fluid”, total urinary volume excretion amounted to 1,897 ± 513 ml by minute 300, while in study group “Zero”, total urinary volume excretion amounted to 679 ± 360 ml (Fig. 2g, Table 4). In study group “Fluid”, body weight increased from the baseline value of 77.4 ± 12.7 kg in minute 0 to the maximal value of 79.0 ± 12.8 kg in minute 60 and remained continuously elevated in comparison to baseline throughout minute 300. In study group “Zero”, body weight decreased from the baseline value of 77.4 ± 12.7 kg in minute 0 to the minimal value of 76.6 ± 12.5 kg in minute 300 (Fig. 2h, Table 4). All time points for body weight differed significantly between the two study groups except at the baseline.

**Table 4:** Change in body weight and urine output during the fluid intervention (“Fluid”) and thirsting (“Zero”).

	Fluid	Zero	
Intravenous fluid volume [ml]	2,199 ± 192	/	
Duration of iv fluid application [min]	63.8 ± 1.8	/	
Body weight baseline [kg]	77.4 ± 12.7	77.4 ± 12.7	p = 0.967
Body weight at 60 min [kg]	79.0 ± 12.8	77.3 ± 12.6	p < 0.001**
Body weight at 300 min [kg]	77.7 ± 12.7	76.6 ± 12.5	p < 0.001**
Urinary excretion at 60 min [ml]	607 ± 296	104 ± 152	p < 0.001**
Urinary excretion at 300 min [ml]	1897 ± 513	679 ± 360	p < 0.001**

All values are means ± standard deviations. **Significant difference between “Fluid “and “Zero” was determined by Student’s t-test at the 0.01 probability level.

Peripheral capillary oxygen saturation, heart rate and blood pressure were measured during the first 90 minutes of the study, as well as at discharge in minute 300 (Fig. 2f, Table 5). Peripheral capillary oxygen saturation was between 98% and 99% at all time points in both study groups. In minute 20, 30 and 70, peripheral capillary oxygen saturation was 1% lower in study group “Zero” than in study group “Fluid” (p < 0.05). In minute 70, respectively minute 90, heart rate in study group “Zero” was 6, respectively 7 beats per minute lower than in study group “Fluid” (55 ± 6 bpm versus 61 ± 10 bpm, respectively 54 ± 8 bpm versus 61 ± 12 bpm, both p < 0.05). Mean blood pressure did not differ between the study groups. From minute 50 to minute 90, systolic blood pressure was higher in study group “Fluid” than in study group “Zero”, (all p < 0.05). The biggest difference in systolic blood pressure was observed in minute 60: systolic blood pressure was 121 ± 10 mmHg in study group “Fluid” versus 112 ± 11 mmHg in study group “Zero”.

**Table 5:** Hemodynamic measurements during the fluid intervention (“Fluid”) and thirsting (“Zero”).

		Baseline	10 min	20 min	30 min	40 min	50 min	60 min	70 min	80 min	90 min	300 min
Heart rate [beats/min]	Fluid	62.8 ± 8.9	58.2 ± 9.2	58.5 ± 9.1	58.1 ± 9.2	56.8 ± 9.8	57.1 ± 9.0	59.1 ± 8.9	**61.3** **±** 9.5**	59.9 ± 9.9	**61.1** **±** 11.9*	59.7 ± 8.3
	Zero	62.7 ± 8.4	59.0 ± 7.1	60.2 ± 7.8	57.9 ± 6.7	57.1 ± 5.6	56.7 ± 7.0	57.7 ± 5.8	**55.1** **±** **6.0**	54.8 ± 8.2	**54.3** **±** **7.6**	57.9 ± 5.4
Systolic RR [mm Hg]	Fluid	119.1 ± 12.1	115.9 ± 10.0	114.7 ± 9.7	117.1 ± 9.6	116.6 ± 10.6	**119.4** **±** 8.9**	**120.9** **±** 9.6**	**121.1** **±** 10.2**	**119.3** **±** 11.9*	**119.1** **±** 11.1*	119.5 ± 10.9
	Zero	118.2 ± 11.7	116.1 ± 12.5	113.8 ± 12.4	114.0 ± 11.9	113.7 ± 11.5	**113.5** **±** **11.1**	**112.1** **±** **11.2**	**113.7** **±** **12.6**	**115.1** **±** **10.2**	**114.8** **±** **10.7**	119.4 ± 10.7
Diastolic RR [mm Hg]	Fluid	64.5 ± 9.8	61.6 ± 10.3	62.5 ± 11.7	64.3 ± 10.0	63.8 ± 10.8	65.1 ± 10.0	65.3 ± 10.1	63.4 ± 8.7	63.3 ± 10.8	64.5 ± 10.6	64.9 ± 11.1
	Zero	64.0 ± 10.5	61.5 ± 11.8	60.8 ± 11.8	62.7 ± 11.2	61.1 ± 10.1	64.1 ± 11.9	63.5 ± 10.4	63.7 ± 10.7	64.7 ± 11.0	64.6 ± 9.7	65.1 ± 8.8
Mean RR [mm Hg]	Fluid	83.4 ± 9.9	79.9 ± 8.6	80.5 ± 9.8	82.6 ± 8.5	81.1 ± 8.2	83.9 ± 9.3	84.0 ± 8.5	82.6 ± 8.3	82.3 ± 9.5	82.6 ± 9.4	84.2 ± 9.7
	Zero	83.3 ± 10.6	80.5 ± 11.3	79.7 ± 12.0	81.5 ± 12.3	80.5 ± 10.7	82.2 ± 11.6	81.1 ± 9.8	82.5 ± 12.2	83.2 ± 10.6	82.8 ± 10.8	81.5 ± 11.5
O_2_ Saturation [%]	Fluid	97.7 ± 1.3	98.4 ± 1.6	**98.7** **±** 1.4**	**99.0** **±** 1.4**	99.0 ± 1.5	98.9 ± 1.2	99.0 ± 1.3	**99.1** **±** 1.3*	98.8 ± 1.6	99.3 ± 1.2	98.8 ± 1.3
	Zero	97.9 ± 0.9	98.0 ± 1.3	**97.7** **±** **1.1**	**98.2** **±** **1.5**	98.5 ± 1.0	98.4 ± 1.4	98.5 ± 1.6	**98.4** **±** **1.5**	98.5 ± 1.2	99.3 ± 1.2	98.7 ± 1.6

All values are means ± standard deviations. Significance was determined by Student’s t-test. **Significant difference between “Fluid “and “Zero” at the 0.01 probability level. *Significant difference between “Fluid “and “Zero” at the 0.05 probability level. Abbreviation: RR = blood pressure. Boldface indicating statistical significance (p < 0.05).

## Discussion

In the present cross-over study, we rapidly infused an “isotonic”, balanced infusion solution into healthy volunteers on study day “Fluid”, and used biompedance spectroscopy to assess their fluid volume changes. After the administration of 2.2 l ELO-MEL-isoton on average within one hour, the fluid volume status underwent clinically meaningful alterations over the short-time infusion period, which remained detectable through the 5-hour observational period. While fluid load, extracellular volume and total body volume increased during the infusion and remained significantly elevated throughout the observational period, compared to baseline and study day “Zero”, intracellular volume decreased during the infusion period and slowly returned to baseline throughout the observational period. Total urinary volume averaged to 1.9 l versus 0.7 l by 5 hours in the “Fluid” versus “Zero” group, respectively, and systolic blood pressure was significantly higher in “Fluid” than in “Zero” between minute 50 and minute 90, when extracellular volume expansion in the “Fluid” group was highest.

Intravenous fluid therapy is not only common^15^, but also appears to be indispensable in severe medical conditions, although the question how to perform adequate fluid resuscitation is perhaps among the ‘hottest topics’ in intensive care medicine. A landmark randomized controlled clinical trial^16^ indicated that protocolized delivery of early, goal-directed therapy (EGDT) to patients presenting to the emergency department with septic shock reduced hospital mortality and hospital stay. EGDT was aimed at achieving a central venous pressure (CVP) of 8–12 mmHg, and based on this study^16^, a CVP target of 8–12 mmHg entered the surviving sepsis guidelines^17^, despite three multicentre trials (ProCESS^18^, ARISE^19^, and ProMISe^20^) after the initial study^16^ showing that EGDT was not superior to usual care. In another study on fluid resuscitation in septic shock, where a positive fluid balance and elevated CVP were associated with increased mortality^21^, the authors emphasized the difficulty of *stopping* the volume infusion, which was not part of the EGDT protocol, and even more drastic terms have been used to describe the dilemma of overloading patients with fluid^22^.

The potential danger of a positive fluid balance has been emphasized in a large pan-European study of critically ill patients, where a positive fluid balance was among the strongest prognostic factors for death^23^, while a much earlier, but very small retrospective study showed that at least one day of negative fluid balance achieved by the third day of treatment may be a good independent predictor of survival in patients with septic shock^24^. A recent randomized, parallel group multicentre feasibility trial in patients with septic shock pointed towards benefit with fluid restriction^25^. In critically ill patients with acute kidney injury, fluid overload was independently associated with mortality^26^. Clearly, for acute lung injury, a positive fluid balance has been shown to increase the time spent on mechanical ventilation and to trend toward increased mortality^27^, while a negative cumulative fluid balance at day 4 was associated with significantly lower mortality^28^. In surgical patients, a restricted intravenous fluid regimen significantly reduced postoperative complications^29^, and vice-versa, a positive fluid balance was associated with complications after elective open infrarenal abdominal aortic aneurysm repair^30^.

While the list of studies reporting a negative impact of fluid overload in the intensive care setting and elsewhere might be continued unceasingly^31–40^, how does the present analysis relate? Intravenous fluid therapy may lead to hospital-acquired generalized interstitial oedema (HAGIE)^41^, but repeatedly, the argument has been brought forth that this is in sick patients with increased vascular permeability, and possibly kidney or heart disease. The fact that an ‘isotonic solution’, infused into healthy males with a normal capillary leak index, leads to a decrease in intracellular volume and fluid accumulation in the extracellular volume, might lead to the conclusion that the infusion itself is responsible for fluid load in the hospital-setting. Importantly however, our healthy volunteers are not an appropriate model for fluid therapy of patients with capillary leak and intracellular derangements form organelle dysfunction, and our results do not imply that we know what the corollary of intracellular fluid shifts and an increase in extracellular fluid really might be.

Clearly, the ideal fluid for resuscitation and other purposes has not been identified^42^. In the past, colloid solutions have been advocated, which, in theory, maintain an effective colloid osmotic pressure or tonicity relative to the extracellular fluid compartment. A systematic (Cochrane) review on colloids versus crystalloids for fluid resuscitation in critically ill patients, however, revealed no evidence that colloids reduce the risk of death^43^. Among colloids, hydroxyethyl starch is even ineligible for volume resuscitation, as its use in the critically ill is associated with an increased risk of mortality and acute kidney injury^44^. Adverse outcomes could be mediated by damage done to the fragile, endothelial glycocalyx layer, which is already at risk in patients with sepsis^45, 46^, undergoes changes through inflammatory and pathological conditions on tissue permeability^47^, and can be further disrupted by rapid intravenous infusion of fluids^48, 49^. In view of the present data and the association between a positive fluid balance with adverse outcomes, management of fluid therapy might need to be reassessed.

In order to rule out that ELO-MEL-isoton was simply the ‘wrong’ infusion solution, while solutions with lower osmolarity might yield different results, a subsequent experiment should be performed with lactated Ringer’s solution (osmolarity = 278 mosmol/l), in comparison with normal saline (osmolarity 308 mosol/l). Alternatively, it might be rewarding to continue searching for a more physiological infusion solution. In addition, treatment with liquids by the oral and gastrointestinal routes of administration should be reconsidered, as in two previous publications^50, 51^, although we acknowledge as previously, that our data from healthy volunteers do not allow a direct jump to this conclusion.

Studying gastrointestinal fluid administration, however, seems logical as the process of adsorbing fluid and electrolytes through the gut potentially leads to a more adequate intravasal osmolar balance than the administration of an ‘unphysiological’ infusion solution, meanwhile keeping the gut active in patients who suffer from illness, who are otherwise deprived from food and fluid intake during the early hospitalization period, especially in intensive care. In following this rationale, it should be remembered that the origins of intravenous fluid therapy date back to the outbreak of cholera, spreading across Asia and Europe from India where it began in 1829^52^. Patients with cholera, however, suffer from diarrhoea and severe dehydration as a consequence of cholera toxin’s activating cystic fibrosis transmembrane conductance regulator channels in the small intestine, which in turn causes sodium and chloride loss from inside the cells. In contrast to patients with cholera, not all hospitalized patients nowadays are severely dehydrated. It therefore lies at hand that fluid might not necessarily have to be administered intravenously.

Returning to the data of the present study, several details merit further discussion. Despite significantly larger urine output after 300 minutes in study group “Fluid”, we also measured a significant weight gain of 0.3 kg at the end of the study day (p = 0.015). This result indicates that fluid actually gets stored, even in healthy subjects. In line, Holte *et al*. reported a persistent median weight gain of 0.85 kg after infusion of 40 ml/kg lactated Ringer’s solution 24 hours later in healthy volunteers^53^. Other studies showed that a saline infusion of 22 ml/kg was not excreted until approximately two days later, again in young healthy volunteers^54, 55^.

Peripheral oxygen saturation was marginally better in study group “Fluid”, compared to “Zero” in minutes 20, 30 and 70, and systolic blood was higher from minute 50–90, while heart rate was lower at several time-points. These results are in accordance with basic physiological principles. An increase in systolic blood pressure can be explained by the Frank-Starling mechanism, as intravenous fluid therapy increases cardiac filling, which improves cardiac output.

Study limitations: The sample size of the present study is small, which represents a study limitation. However, the results of our analysis are likely solid, because the direction of the data points was consistent. Applying a multiple testing procedure would not change the inference from our data analysis: The differences of the area under the curve for fluid load, relative fluid load and extracellular volume between the two groups remain highly significant even after Bonferroni correction for having tested five different outcome variables.

We were unable to compare our BCM results with additional reference methods. Using the BCM, bioelectrical properties are measured between wrist and ankle and the three body segments arms, trunk and legs with different cross sectional areas and lengths influence whole body impedance. While the limbs add 90%, the trunk contributes 10% to the impedance^56^. Whole body bioimpedance spectroscopy seems to be more sensitive in volume changes in the limbs, and therefore, extracellular fluid accumulation in the trunk could be incompletely measured by this method^57^. Although we cannot formally rule out that bias might have been introduced by the method itself, the BCM had been validated against direct estimation methods in more than 500 healthy subjects^8, 10^, and is accepted as a very precise whole body bioimpedance spectroscopy device^58–61^.

It has been recommended that subjects should be resting in a supine position for at least 5 minutes, before the BCM-measurement is started, such that fluid volume equilibration has taken place. The spikes in fluid load, relative fluid load and extracellular volume that were observed after the study volunteers rose up to urinate (Fig. 2a, c,e) may be reflective of disequilibration. These spikes might have been amplified because the body weight was entered precisely at each BCM-measurement.

In conclusion, when we tested the efficacy of whole-body bioimpedance spectroscopy for assessment of fluid volume status at baseline, as well as for detection of rapid and longer-term changes in fluid load, extracellular volume, extracellular volume, intracelluIar volume and total body volume under standardized, “isotonic” intravenous fluid therapy, we observed a statistically significant, and clinically meaningful decrease in intracellular volume, and an increase in extracellular volume throughout the study period. We posit that the observed fluid volume changes, which occurred in perfectly healthy subjects, may be useful in searching for explanations of the adverse effects that have been reported for fluid therapy in the clinical setting. The present results may moreover inspire our quest to search for a more physiological infusion solution, and to test the gastrointestinal route of fluid administration, even in patients with critical illness.

## Materials and Methods

### Study Participants, Design and Procedures

Study participants had to volunteer, and had to be healthy males, ≥18 years of age. Criteria for health included absence of adiposity (body mass index ≥30 kg/m^2^), absence of hypotension (systolic blood pressure ≤90 mmHg) and hypertension (systolic blood pressure ≥160 mmHg, both values obtained after 5 minutes of resting), absence of cardiac insufficiency (NYHA stage ≥2)^62^ and absence of renal insufficiency (estimated glomerular filtration rate <60 ml/min/1.73 m^2^ [MDRD-IDMS formula]). Participation in another clinical trial within the last 4 weeks prior to study day 1 precluded the study participation. All participants were recruited from the database of the Medical University of Vienna’s Department of Clinical Pharmacology from October 2014 until March 2016. Initial contact with the participants was established by telephone or mail, to inquire about their interest and availability. Written informed consent was obtained prior to study day 1, and blood was drawn immediately thereafter for analysis of laboratory values at baseline (blood cell count, haemoglobin, C-reactive protein [CRP], serum protein and albumin, oncotic pressure, renal function [serum creatinine and estimated glomerular filtration rate], hepatic function [gamma glutamyl transferase]). Capillary leak index (CLI) was calculated as the ratio of CRP (in milligrams per decilitre) over serum albumin (in grams per litre), multiplied by 100, as a parameter for vascular permeability.

The present study followed a randomized, crossover design, complied with the Declaration of Helsinki and was approved by the local ethics committee (The Ethics Committee of the Medical University of Vienna # 1429/2014) before registration with ClinicalTrials.gov (NCT02296294, 18/11/2014). Randomization into the study group “Fluid” or study group “Zero” was performed on study day 1, using sealed, opaque envelopes. In study group “Fluid”, participants received intravenous fluid therapy with the ELO-MEL-isoton infusion solution (Fresenius Kabi, Austria, osmolarity = 302 mosmol/l, content [in mmol/1,000 ml]: Na^+^ = 140, K^+^ = 5.0, Ca^++^ = 2.5, Mg^++^ = 1.5, Cl^−^ = 108.0, Acetate = 45.0) at a rate of 0,50 ml/kg/min (kg ideal body weight) for 60 minutes. The well-validated^63^ Robinson Formula^64^ was used for calculation of the ideal body weight^31^ = (Height [cm] −154,2)/2.54 × 1.9 + 52. In study group “Zero”, participants received no therapy at all, and had to remain thirsting. After completion of study day 1, all participants crossed over into their respective remaining study group, for the second study day (Fig. 1).

On the morning of both study days, participants were admitted to the clinical research ward of the Medical University of Vienna after having fasted 6 hours prior. Participants urinated before determination of the baseline weight. Physical status was recorded using the American Society of Anaesthesiologists’ classification system^65^ and the NYHA functional classification^62^. Participants occupied a supine position, and were monitored for oxygen saturation (SpO_2_) and non-invasive blood pressure (NIBP), as well as by electrocardiogram (ECG), at baseline and in 10-minute intervals thereafter for 90 minutes. BCM-measurements were also performed at baseline and in 10-minute intervals thereafter for 300 minutes, and BCM-electrodes were taped for the whole study day. In the “Fluid” group, a plastic cannula (Venflon, size 16 Gauge) was inserted into an antecubital vein of the arm contralateral to the BCM-measurement. The infusion solution was started immediately thereafter.

After the start of the study clock, participants urinated every 60 minutes into a urine receiver, and were reweighed immediately thereafter. The supine position was abandoned during this process. All urine volumes were measured from minute 60 onwards. The entire study duration was 300 minutes on both study days.

### BCM-measurements

The BCM is a whole-body bioimpedance spectroscopy device, manufactured and distributed by Fresenius Medical Care (FMC) Germany^61^. This device was originally designed to determine the fluid volume status including fluid load (termed fluid overload on the BCM-device) in patients with end stage renal disease on renal replacement therapy, thereby aiding the optimal fluid removal during the dialysis session (the ‘dry weight’ prescription)^39^. Briefly, the BCM determines resistance and reactance at 50 discrete frequencies from 3 to 1,000 kHz. Extracellular and intracellular resistance are obtained on the basis of a Cole model^66^. Using these read-out values, extracellular volume, intracellular volume, and total body volume are automatically calculated by the BCM-device, employing a fluid model described by Moissl^10^. Subsequently, excessive extracellular fluid is distinguished from the hydration of major body tissue, on the basis of a physiological tissue model described by Chamney^8^. The latter model provides the normal hydration status for a human being of given weight, height and sex, i.e. the expected normal values for extracellular volume and intracellular volume that would usually occur in an individual with adequate renal function, and in a state of normohydration. The actual tissue hydration is then calculated from the difference between the normal and the measured values for extracellular volume and intracellular volume. Excess extracellular fluid is provided as ‘fluid overload’ or ‘overhydration’ in litres, and in % above “normal” extracellular volume, as a numerical value on the BCM-device. In dehydrated patients, the value for ‘overhydration’ on the BCM-device reads negative.

BCM-measurements are painless, requiring the taping of 4 non-recyclable BCM-electrode strips to wrist and ankle, respectively hand and foot, on one side of the body. The results of the BCM-measurements are automatically stored on a patient BCM-card. Besides fluid volume status, a typical BCM-measurement also provides more basic information on the body composition, such as lean tissue mass, lean tissue index (=lean tissue mass/height^2^), adipose tissue mass, fat tissue index (=adipose tissue mass/height^2^), and fat mass. Bioimpedance spectroscopy has high reproducibility and sensitivity^67^. Extensive validation of the fluid volumes and body composition has been performed against reference methods (bromide-, deuterium dilution and total body potassium method), involving healthy volunteers and haemodialysis patients^8–10^.

A technical note: The terms fluid overload, volume overload and overhydration are often used interchangeably. Since ‘hydration’ refers strictly to water, while ‘volume expansion’ refers to the accumulation of isotonic fluid (salt and water), we primarily used the term fluid load and fluid status for our study in healthy volunteers. Similarly, the term extracellular water used in previous publications^38, 68–70^ has here been replaced by extracellular fluid volume.

### Statistics and Sample Size Considerations

Descriptive statistics (mean, variance, proportion; and median, 25^th^ and 75^th^ percentile [where appropriate]) were used to portray the participants’ characteristics, laboratory values, hemodynamic measures and BCM-results. The primary statistical analysis was performed for the 5 following variables: fluid load, relative fluid load in % extracellular volume, total body volume, extracellular volume and intracellular volume. (Note that relative fluid load [%] = fluid load/extracellular volume × 100; total body volume = extracellular volume + intracellular volume.) These 5 variables were measured every 10 minutes over a period of 300 minutes. Data were systematically missing in minute 200 and in minute 260. For 9 patients, data were missing at random for different time points. Imputation was performed in almost all cases by linear interpolation (taking the mean of the neighbouring data points) except for one patient who had missing data at the final measurement (in minute 300). In the latter case, a simple last observation carried forward strategy was applied and the values from minute 290 were used for imputation. For the imputed data the area under the curve (AUC) was calculated using the trapezoidal rule for all 5 variables. Statistical data analysis for the cross-over trial was performed using for each variable a mixed model (SAS PROC MIXED) with “treatment” and “study day” as fixed effect and “patient” as random effect^71^. We reported p-values without correcting for multiple testing. Simple Bonferroni correction can be applied in discussing the results if one wanted to take into account that five variables were tested.

To illustrate the longitudinal data sets we calculated average profiles for the two treatment groups including pointwise standard errors of the mean. To illustrate substantial differences for fluid load, extracellular volume, intracellular volume and total body volume between “Fluid” and “Zero” at the various time-points, we also used the two-sided Student’s t-tests in an illustrative, exploratory fashion.

Additionally, we calculated the variable “completion” and “Δ Baseline-to-completion” for all BCM derived fluid volume measurements. “Completion” represented the mean from minute 240 to minute 300 and respectively, “Δ Baseline-to-completion” the mean difference from baseline to completion.

Sample size calculation was based on the hypothesis that routine perioperative iv fluid administration might lead to an increase in fluid load and extracellular volume, even in healthy volunteers. In our previous observational study in 71 gynaecological patients, a net perioperative fluid balance of 1.6 ± 0.7 l had led to an increase of 0.8 ± 0.8 l in fluid load (0.8 ± 0.7 l in extracellular volume)^11^. Regarding the present crossover study, we used our previous data, but derived a hypothetical 2-group comparison. Assuming two-sided testing and a standard deviation of 1 l, α = 0.05 and β = 0.2, a sample size of 14 participants was chosen to detect a between-group difference of at least 1.5 l in fluid load, which we considered clinically meaningful. The sample size was increased to 15 participants, to account for potential drop-outs.

The Statistical Package for Social Sciences (SPSS 20.0) and Microsoft Excel 2010 was used for the analyses mentioned above.

### Data availability

All datasets generated during the current study are available from the corresponding author on reasonable request and the results from all data analysed during this study are included in the published article.
